# Fronto-medial electrode placement for electroconvulsive treatment of depression

**DOI:** 10.3389/fnins.2022.1029683

**Published:** 2022-10-20

**Authors:** J. Douglas Steele, Tom Farnan, David M. Semple, Siwei Bai

**Affiliations:** ^1^School of Medicine, University of Dundee, Dundee, United Kingdom; ^2^University Hospital Hairmyres, NHS Lanarkshire, Glasgow, United Kingdom; ^3^Department of Electrical and Computer Engineering, Technical University of Munich, Munich, Germany

**Keywords:** ECT, side-effects, electric fields, depression, computational modeling

## Abstract

Electroconvulsive therapy (ECT) is the most effective treatment for severe treatment-resistant depression but concern about cognitive side-effects, particularly memory loss, limits its use. Recent observational studies on large groups of patients who have received ECT report that cognitive side-effects were associated with electric field (EF) induced increases in hippocampal volume, whereas therapeutic efficacy was associated with EF induced increases in sagittal brain structures. The aim in the present study was to determine whether a novel fronto-medial (FM) ECT electrode placement would minimize electric fields in bilateral hippocampi (HIP) whilst maximizing electric fields in dorsal sagittal cortical regions. An anatomically detailed computational head model was used with finite element analysis, to calculate ECT-induced electric fields in specific brain regions identified by translational neuroimaging studies of treatment-resistant depressive illness, for a range of electrode placements. As hypothesized, compared to traditional bitemporal (BT) electrode placement, a specific FM electrode placement reduced bilateral hippocampal electric fields two-to-three-fold, whilst the electric fields in the dorsal anterior cingulate (dAC) were increased by approximately the same amount. We highlight the clinical relevance of this specific FM electrode placement for ECT, which may significantly reduce cognitive and non-cognitive side-effects and suggest a clinical trial is indicated.

## Introduction

Psychiatric illnesses are the leading cause of long term disability world-wide with depression the commonest cause (Whiteford et al., 2013; WHO, 2018). Better treatments, with improved efficacy and reduced side-effects, are needed. Electroconvulsive therapy (ECT) is the most effective treatment for severe treatment-resistant depression, typically used when patients have not responded to multiple treatments or need a rapid response with faster action than other treatments (Pagnin et al., 2004; Wade et al., 2020).

A recent report from the Scottish ECT Accreditation Network (SEAN) national audit (SEAN, 2021), covering a decade of treatments from 2009 to 2018, with ∼4000 treatments per year, found the majority of patients receiving ECT were in the 50–79 years old age range, the most common diagnosis was depression with or without psychotic symptoms, ∼15% of treatment episodes were recorded as emergency lifesaving treatments, and 75% of patients were “much improved” or “very much improved” after a course of ECT. The commonest reported side-effects in the SEAN reports were memory problems, headache and muscle aches and brief disorientation.

Side-effects on memory are among the most important factors limiting acceptance, prescription and use of ECT and may contribute to premature discontinuation of treatment, or a lack of use of ECT when it could be effective (Finnegan and McLoughlin, 2019). Whilst there is little evidence for anterograde memory impairment persisting for longer than a few weeks after the last treatment, the duration of retrograde amnesia is more controversial. In practice, minimization of cognitive side-effects is by identification of patients at higher risk and modifying treatment delivery: e.g., stimulus dose, frequency of treatments, pulse-width, and electrode placement. Bitemporal (BT) placement of electrodes has the greatest therapeutic efficacy but most cognitive side-effects; high dose right unilateral (RUL) electrode placement has fewer side-effects (Finnegan and McLoughlin, 2019) but is generally regarded as less effective.

In health, the hippocampus is crucial for human episodic memory encoding (Squire, 1986; Lisman et al., 2017) with the anterior region also implicated in emotional experience (Gray and McNaughton, 2000; Phelps, 2004). Adverse memory side-effects of ECT are thought to be predominately related to stimulation of the hippocampus, although other regions including the inferior and middle frontal gyri are important for memory retrieval, with all regions stimulated to a variable extent by a wide-variety of ECT electrode placements (Bai et al., 2019). However, for patients who have not received ECT, depressive illness is itself associated with cognitive, including memory impairment (Anderson et al., 2021), and there is robust evidence for hippocampal volume reduction in unipolar and bipolar illnesses (Hibar et al., 2016; Schmaal et al., 2016).

Recently, a series of publications from the Global ECT-MRI Research Collaboration (GEMRIC) have reported important findings. First, a longitudinal neuroimaging study of brain structure in a large number of patients reported that the number of ECT sessions and electrode placement impacts the extent and laterality of ECT-induced hippocampal enlargement, but there was no relationship between hippocampal volume enlargement and clinical outcome (Oltedal et al., 2018). A second study calculated the electric field (EF) induced by ECT in different brain regions, reporting a strong relationship between EF intensity and brain volume increase, but that neither structural volume increase nor EF intensity were associated with antidepressant response (Argyelan et al., 2019) although a relationship has also been reported (Deng et al., 2021). These were univariate studies and an alternative multivariate approach can be helpful (Steele and Paulus, 2019). A third GEMRIC study tested for associations between 80 different brain regions and clinical outcome using a multivariate approach, reporting that structural brain increases in cortical mid-line structures, such as the dorsal anterior cingulate (dAC) and precuneus posterior cingulate (PPC), predicted individual patient response but not medial temporal regions, such as the amygdala and hippocampus, which have been the focus of ECT research thus far (Mulders et al., 2020). Its long been established that induction of a generalized seizure is required for efficacy, and these recent studies suggest that an ECT electrode configuration specifically designed to maximize the induced EF in dorsal sagittal structures, simultaneously minimizing the induced EF in bilateral HIP, could be optimally therapeutic and minimize cognitive side-effects.

Electric field is typically maximal in brain regions immediately deep to the ECT electrodes. This means an electrode placement rotated through 90 degrees from the ubiquitous (in the UK) BT orientation, with the anterior electrode near the dAC and posterior electrode near the PPC in a fronto-medial (FM) configuration (Lee et al., 2016), should maximize the induced EF in these structures, also maximizing the distance from the electrodes to bilateral HIP. However, the precise EF in specific brain sub-regions identified as important in clinical neuroimaging studies of treatment-resistant depression is unclear. Also, different FM electrode placements are possible. Therefore, we used a computational model (Bai et al., 2019) to test the hypothesis that an optimal FM placement would maximize the EF in specific sagittal brain regions whilst minimizing the EF in subregions of bilateral HIP, identified by neuroimaging studies of patients with treatment-resistant depression who had not received ECT.

## Materials and methods

### Head computational model and analyses

A finite element head model was constructed from a 3T MRI T1-weighted 1 mm isotropic resolution brain scan provided by the NIH Human Connectome Project (MGH1010, gender: female, age: 25–29) (Fan et al., 2016). Briefly, head tissue compartments were analyzed using Brainsuite^1^ to segment skin and subcutaneous tissue (fat and muscle), skull, cerebrospinal fluid, gray matter, and white matter. Segmented tissue compartments were then imported into 3D Slicer^2^ for manual correction of voxels as required (Bai et al., 2019; Bakir et al., 2019). The tissue compartments were assumed to be homogeneous with conductivities assigned from the mean values from multiple studies (Bai et al., 2019). Modeling was done using COMSOL Multiphysics (v5.0) finite element analysis software (COMSOL AB, Stockholm, Sweden) with a direct linear solver. Mean and standard deviation of the EFs were calculated from 100 points selected randomly within anatomically defined regions of interest (ROI).

### Regions of interest for electric field and current density calculations

**FIGURE 1:**
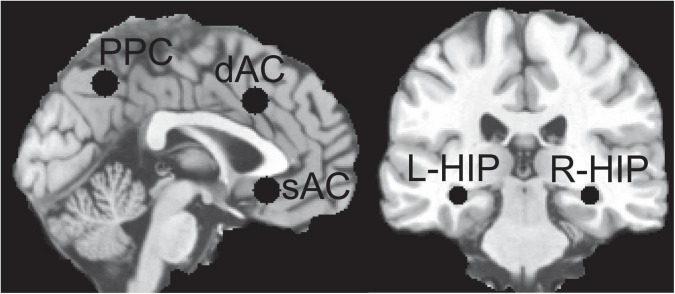
Regions of Interest (ROI) used for sampling the electric field (EF) distributions; left hippocampus (L-HIP), right hippocampus (R-HIP) and for other abbreviations see main text.

We calculated EFs in specific sagittal brain regions and bilateral HIP for different electrode configurations. *A priori* defined spherical ROI were defined using Montreal Neurological Institute (MNI) standard brain anatomy selected from the translational neuroimaging studies discussed later: dAC (0,19,37) 15 mm (Steele et al., 2008; Tolomeo et al., 2016), subgenual AC (sAC) (0,25,−9) 15 mm (Drevets et al., 1997), precuneus posterior cingulate (PPC) (0,−58,46) 15 mm (Johnston et al., 2015) hippocampi (HIP) (−32,−28,−10) 10 mm, (34,−26,−10) 10 mm (Johnston et al., 2015), where the coordinate is the location of the center of the sphere and diameter is in mm. These ROIs are shown in Figure 1. The head model segmentation, manual editing, and configuration of COMSOL for electrode placements and solving was done using the scan in its original non-MNI patient anatomical space. To determine MNI equivalent locations, the T1 scan was spatially normalized using SPM12^3^, then the resultant deformation field used to calculate the inverse transformation (Ashburner, 2016), from MNI back to original patient scan anatomical space.

### Electroconvulsive therapy electrode placements

**FIGURE 2:**
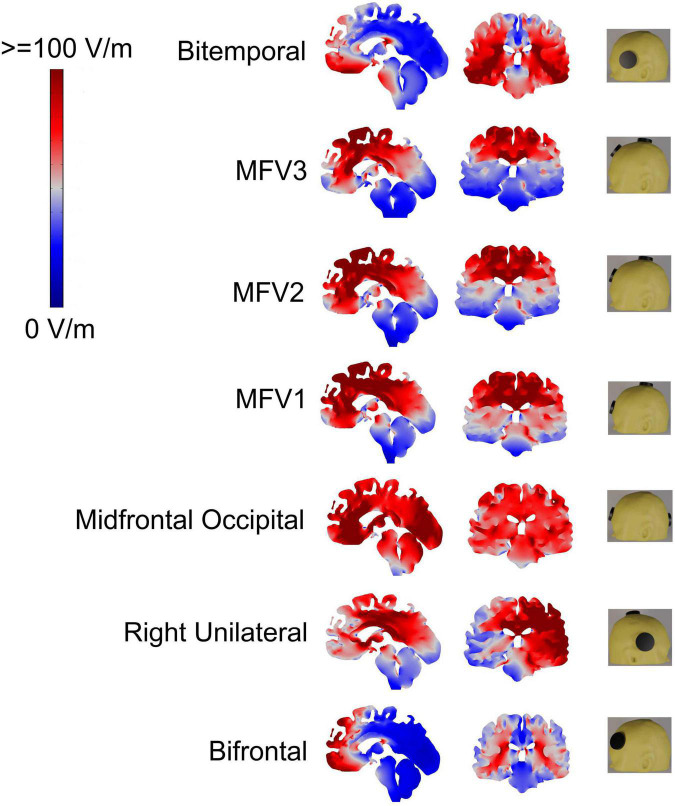
Electric field (EF) distributions and electroconvulsive therapy (ECT) electrode placements; for the latter anterior is to the left except for the right unilateral (RUL) placement, see main text for abbreviations. High EF (red) and low EF (blue) with color bar showing EF in V/m.

A total of 7 bipolar ECT electrode placements, including 4 alternative FM placements, were investigated: (i) BT–center of each electrode was 3 cm superior to the midpoint of a line from the lateral canthus of the eye to the external ear canal, (ii) bifrontal (BF)–center of each electrode was 5 cm superior to the lateral canthus of the eyes, (iii) RUL–anterior electrode 5 cm superior to the lateral canthus of the right eye with posterior electrode just right of the vertex, (iv) mid-frontal-occipital (MFO)–anterior electrode just above nasion and the posterior electrode over the occiput (15), (v) mid-frontal-vertex (MFV1)–anterior electrode just above nasion (as MFO) but with posterior electrode at the vertex, (vi) MFV2–anterior electrode higher at 2.5 cm above nasion and the posterior electrode at the vertex, and (vii) MFV3–anterior electrode highest at 5 cm above the nasion and the posterior electrode at the vertex. MFV3 is the most dorsal of the 4 sagittal FM electrode configurations. These electrode placements are shown in Figure 2 and modeling for each assumed an ECT current of 800 mA delivered to the scalp from a pair of circular electrodes with 5 cm diameter (Bai et al., 2019).

## Results

Figure 2 shows sagittal and coronal views of the EF magnitudes for the 7 electrode configurations with the coronal views chosen to intersect the hippocampi ROIs. As expected, the EFs were highest in brain regions under the electrodes. Mean and standard deviation EFs for selected ROI are shown in Table 1.

**TABLE 1:** Electroconvulsive therapy (ECT)-induced electric fields in V/m (standard deviation) for different electrode placements sampled within specific regions of interest (ROI).

	Left hippocampus	Right hippocampus	dAC	sAC	PPC	dAC/Left hippocampus	dAC/Right hippocampus
BT	99 (16)	84 (19)	33 (12)	53 (7)	26 (2)	0.3	0.4
RUL	59 (8)	82 (10)	75 (19)	72 (18)	76 (8)	1.3	0.9
BF	62 (12)	55 (14)	43 (13)	51 (7)	19 (2)	0.7	0.8
MFO	69 (7)	75 (6)	87 (6)	118 (13)	88 (5)	1.2	1.2
MFV1	56 (6)	59 (7)	113 (17)	101 (14)	80 (8)	2.0	1.9
MFV2	47 (5)	50 (6)	114 (16)	91 (14)	72 (6)	2.4	2.3
MFV3	38 (4)	41 (4)	97 (11)	71 (11)	62 (6)	2.6	2.4

### Electrode placements commonly used in clinical practice

The BT placement resulted in the highest bilateral hippocampal ROI EFs, with high EFs throughout both temporal lobes and particularly high EFs in bilateral temporal lobe white matter. The right hippocampal ROI EF for RUL placement was comparable to that obtained for the BT placement. With a longer distance between the electrodes and hippocampi, BF resulted in lower hippocampal ROI EFs than BT, and significant EFs only in the most anterior medial frontal brain regions. BT resulted in the lowest EF in the dAC ROI, compared to RUL and BF configurations. In contrast, RUL resulted in a high dAC ROI EF because with RUL, the posterior electrode is close to the vertex. Table 1 also shows the ratio of EFs for dorsal AC ROI to each hippocampal ROI: BT had the lowest and RUL the highest ratios.

### Alternative novel sagittal electrode placements

Mid-frontal-occipital had the electrodes placed lowest in the sagittal plane and resulted in the highest bilateral hippocampal EFs for all sagittal placements with significant EFs throughout most of the brain. Moving the posterior electrode from the occipital region to the vertex (MFV1) resulted in a reduction in hippocampal ROI EFs and the EFs throughout both temporal lobes. Then, moving the anterior electrode more superior (MFV2) resulted in a further reduction in hippocampal ROI EFs due to increased distance from the electrodes to both HIP. Hippocampal EFs were minimized by moving the anterior electrode still more superior (MFV3) but this resulted in only a small reduction in the dAC ROI EF, compared with the MFV2 placement. The highest dAC ROI to hippocampal EF ROI ratios were obtained using the most superior MFV3 placement but the increase was marginal in comparison to the MFV2 placement.

It is important that the minimum distance between the two electrodes is not extremely low, to avoid superficial shunting of current between the electrodes due to the electrically resistant skull. BF electrode placement is a long-established method for delivering ECT. Traditional BF electrode placement is 5 cm superior to the outer canthus of the eye, making the distance between the centers of the electrodes about 18 cm or less depending on head size. Deducting 2–3 cm from each of the electrodes due to their size makes the closest edge to edge electrode distance 12–14 cm for BF.

With regard to MFV2 placement and assuming standard EEG positions, then nasion to inion is measured for each patient and percentages are used. By definition Fp1 is 10% from nasion to inion, and the vertex is 50% from nasion to inion. With most patients the nasion to Fp1 distance is about 5 cm, so as Fp1 to vertex is 40% of the distance, the Fp1 to vertex distance is about 20 cm. If we assume the center of the anterior MFV2 electrode is *at Fp1 or no higher than 1 cm above this*, with the posterior electrode at the *vertex*, then allowing for 5 cm diameter electrodes, this makes the minimum MFV2 electrode edge distance 14–15 cm, which compares favorably with BF placement.

Therefore, from the perspective of maximizing the dAC/hippocampus ratios and ensuring an inter-electrode edge distance no less than BF, the optimal FM electrode placement is MFV2.

## Discussion

Electroconvulsive therapy is the most effective treatment for severe and treatment-resistant depression but use is limited because of concern about side-effects (Finnegan and McLoughlin, 2019). ECT causes increases in the volume of brain structures (Argyelan et al., 2019) and ECT-induced increases in the volume of the dentate gyri of the HIP are associated with increased memory impairment (Gbyl et al., 2021). Volume increases in the HIP are not associated with clinical response (Oltedal et al., 2018; Mulders et al., 2020), but increases in the volume of sagittal cortical regions, such as the dorsal anterior and posterior cingulate are associated with clinical response (Mulders et al., 2020). We therefore tested the hypothesis that one of the FM electrode configurations, but not alternative electrode placements such as BT, would minimize the induced EF in the bilateral HIP and maximize the EFs in the dAC and PPC. Detailed computational modeling confirmed this hypothesis.

A large systematic review and meta-analysis of studies of healthy subjects concluded that a sub-region of the dorsal medial cortex, the dAC, integrates subjective experience of negative affect and pain and is important for cognitive control (Shackman et al., 2011). Clinically, anterior cingulotomy, which comprises small bilateral lesions in the dAC, has long been used as a treatment of last resort for severe treatment-resistant depressive illness, when all other treatments including ECT have been tried and failed, the patient wants the operation and can provide sustained informed consent (Steele et al., 2008). In an observational study of patients who had received cingulotomy, we reported the dAC has a causal role in negative affect so lesions in this region could be therapeutic for patients with otherwise intractable mood, anxiety and pain syndromes (Tolomeo et al., 2016). These findings for cingulotomy may be consistent with a study on different patients who had received ECT and not surgery, where we reported ECT decreases the connectivity between the dAC and other brain regions (Perrin et al., 2012). An unexpected result was that smaller dAC lesions were associated with better clinical response (Steele et al., 2008), suggesting the therapeutic effect may be due to smaller lesions inducing connectivity and plasticity change. Consistent with this, ECT increases plasticity (Dukart et al., 2014) and changes in the dAC predicted clinical response (Mulders et al., 2020).

Depressive illness has been associated with abnormal function of other cingulate regions. We and others reported that the sAC and rostral AC have a blunted reward response using fMRI (Johnston et al., 2015; Rupprechter et al., 2020) with reduced connectivity from the sAC-rostral AC to the basal ganglia (Rupprechter et al., 2020). Others have reported sAC functional abnormalities using PET (Drevets et al., 1997; Mayberg et al., 1999) with a small deep brain stimulation trial reporting beneficial effect (Mayberg et al., 2005) but a larger blinded study did not report efficacy (Holtzheimer et al., 2017). Using fMRI, we also reported blunting of the reward response of the PPC of patients with treatment resistant depression (Johnston et al., 2015), and abnormally increased connectivity between the PPC and the lateral orbitofrontal cortex has been reported (Cheng et al., 2018). Notably, the MF electrode configuration also increases ECT-induced EFs in the sAC and PPC regions compared to alternative configurations such as BT, implying it could induce gray matter increase and plasticity also in these regions.

There is robust evidence from two large ENIGMA consortium studies for hippocampal volume reductions in unipolar (Schmaal et al., 2016) and bipolar (Hibar et al., 2016) illnesses. An influential review of preclinical studies predicted that depression is associated with hippocampal abnormalities linked to ruminations (Deakin and Graeff, 1991; Deakin, 2013) and our fMRI study on patients with treatment-resistant depression reported consistent hippocampal abnormalities (Johnston et al., 2015). Cognitive abnormalities in patients with depression who have not received ECT may be linked to abnormal hippocampal function (Johnston et al., 2015). However, whilst ECT is associated with hippocampal volume increases, reversing depression-associated hippocampal decreases, there is clinical evidence that this change does not represent a causal therapeutic effect (Oltedal et al., 2018; Argyelan et al., 2019) although a relationship has also been reported (Deng et al., 2021). Antidepressants also cause neurogenesis and hippocampal volume increase (Malberg et al., 2021) but without adverse effects on memory (BNF, 2016) and some effective treatments (Steele et al., 2008) have no direct effect on the hippocampus. In contrast to uncertainty about ECT therapeutic mechanisms, evidence for ECT-induced memory problems is relatively robust, particularly for BT (Finnegan and McLoughlin, 2019) which we show is associated with the highest bilateral hippocampal ECT-induced EFs. Alternative electrode placements such as RUL have been proposed to minimize effects on one hippocampus, with RUL treatment efficacy comparable to BT by administering RUL ECT at six times the threshold dose (Finnegan and McLoughlin, 2019). Notably though, we found the induced EF in the stimulated hemisphere with RUL is as high as BT assuming the same stimulation parameters. This implies that in clinical practice the right temporal lobe and hippocampus receives a higher EF with RUL placement than with BT, but the left hemisphere a low EF presumably accounting for reduced cognitive side-effects overall.

Previous studies have used EF modeling to investigate different electrode placements, including BT, BF, RUL, and other configurations including MFO, which all are associated with significant ECT-induced hippocampal EFs (Bai et al., 2019). EF calculations for different electrode placements which included a version of FM have also been reported, which are consistent with our findings. However, the aims of these earlier studies were different: determination of minimum current to induce a seizure in rhesus macaque monkeys using ECT and magnetic seizure therapy using a variety of electrode placements (Peterchev et al., 2015), determination of the spatial extent of brain stimulation necessary to produce a seizure in macaque monkeys using a variety of electrode placements (Lee et al., 2017), and a study that concluded that differences in EF strength from different electrode placements may be linked to cognitive side-effects (Lee et al., 2016). Regarding patients who have actually received a version of FM ECT, to our knowledge there is only a conference abstract published a decade ago reporting a single patient (Rosa et al., 2012); a 40 years old woman with manic phase treatment-resistant schizoaffective disorder, who received as an open label trial, 10 sessions of low current amplitude ECT. The authors reported motor and EEG seizures were qualitatively like conventional ECT, the patient had no significant memory complaints although formal testing was not performed, she had resolution of psychotic and euphoric symptoms, and the authors concluded further investigation of FM placement was indicated.

Our work is novel because (i) it additionally builds on recent GEMRIC (Oltedal et al., 2018; Argyelan et al., 2019; Mulders et al., 2020) studies of patients who have received ECT, (ii) it uses for quantification MNI defined regions reported from neuroimaging studies of patients with severe treatment-resistant depression who have not received ECT (Steele et al., 2008; Johnston et al., 2015; Tolomeo et al., 2016), (iii) we compared four alternative FM electrode placements, and (iv) we highlight the particular clinical relevance of the MFV2 FM electrode placement for actual treatment. This FM electrode placement may have additional clinical advantages over BT electrode placement, by reducing the likelihood of non-cognitive side-effects caused by direct electrical stimulation over temporalis muscle with supraphysiological bite induction, which can cause damage of the tongue, teeth and dental implants, jaw pain and headache (Haghighi et al., 2016; Mulder and Grootens, 2020). Consequently, we suggest a randomized blinded clinical trial is indicated, to compare the therapeutic efficacy and side-effects of MFV2 vs. other placements. Limitations of EF modeling for ECT research have been discussed (Sartorius, 2022), which imply that EFs to brain structures distant from electrodes may be less than calculated, supporting the rationale for the FM orientation avoiding bilateral hippocampal EF effects.

In conclusion, ECT is the most effective treatment for severe treatment-resistant depression yet concern about cognitive side-effects, particularly memory loss, limits prescription and use of ECT (Finnegan and McLoughlin, 2019). We have modeled the induced EF from a range of electrode placements in specific brain regions informed by a range of translational neuroimaging studies, including four alternative FM placements, and highlight the MFV2 placement as being particularly important from a clinical perspective. We predict this electrode placement will minimize cognitive side-effects such as memory loss and may reduce non-cognitive side-effects such as oral cavity damage and headache, whilst maintaining or increasing therapeutic efficacy.

## Data availability statement

Publicly available datasets were analyzed in this study. This data can be found here: NIH Human Connectome Project (MGH1010).

## Ethics statement

Ethical review and approval were not required for the study on human participants in accordance with the local legislation and institutional requirements. Written informed consent was not required for the study on human participants in accordance with the local legislation and institutional requirements.

## Author contributions

JS, TF, and SB conceived the project. SB and TF did the computational modeling. JS wrote the initial draft. All authors discussed the results and contributed to the final manuscript.

## Footnotes

1
http://brainsuite.org/

2
www.slicer.org

3
https://www.fil.ion.ucl.ac.uk/spm/software/spm12/
